# A four-track perspective for bottom-up synthetic cells

**DOI:** 10.3389/fbioe.2022.1029446

**Published:** 2022-09-30

**Authors:** Pasquale Stano

**Affiliations:** Department of Biological and Environmental Sciences and Technologies (DiSTeBA), University of Salento, Lecce, Italy

**Keywords:** artificial cells, synthetic cells, protocells, bottom-up synthetic biology, cell-free systems, sciences of the artificial, wetware artificial life

## “Synthesizing life”, 20 years later

A crucial move for the development of the bottom-up synthetic biology (SB) branch took place about 20 years ago, when Jack W. Szostak, David Bartel and Pier Luigi Luisi co-authored a *Nature* paper entitled “Synthesizing Life” (Szostak et al., 2001), which can be considered a sort of foundational paper for (or even the manifesto of) the modern approaches for constructing living artificial cells from scratch. Possibly as a sign of the *Zeitgeist*, the article was published almost simultaneously to two other foundational papers in SB (Elowitz et al., 2000; Gardner et al., 2000).

The very idea of synthesizing life—the Faustian dream of all times—is not new. Several (unsuccessful) attempts to build cell-like systems of minimal complexity fill the annals of science (Hanczyc, 2009). These attempts share a common anti-vitalistic viewpoint: synthesizing (cellular) life from scratch should be possible, and it would demonstrate that the biological phenomenology follows a “continuity principle” with respect to physics and chemistry. That is, life is an emergent property of some molecular systems characterized by a very peculiar type of structural and dynamical (self-)organization. However trivial and generally taken for granted by scientists, the emergence of life from inanimate matter has never been demonstrated experimentally and it is still one of the big targets in science.

What is, then, the remarkable and novel element that has been put forward in the “Synthesizing Life” article, and that can be considered as a foundational concept for bottom-up approaches in SB? The Authors actually focused on the hypothetical construction of primitive cell (protocell) models, made of catalytically active RNAs (ribozymes) (Bartel and Szostak, 1993; Eckland et al., 1995), encapsulated inside fatty acid vesicles (Hargreaves and Deamer, 1978; Bachman et al., 1992; Walde et al., 1994). The claim is that such structures would display minimal life-like behavior (reproductive and potentially evolutive) if the intravesicle ribozymes catalyze their own replication and the production of membrane molecules at the expenses of certain precursors available in the environment. The whole process would lead to a spontaneous growth-division of protocells in an allegedly primitive Earth scenario.

Leaving aside, for the moment, the mechanistic details and their plausibility, the fundamental and explicit message of that paper goes beyond the apparently narrow focus on the origin of life. To a closer inspection, in fact, the Authors put forward an operational methodology for the construction of chemical reacting systems that would show the difficult-to-define property of being alive just by fulfilling a specific structural and dynamic organization. The latter is described by features as: (1) self-bounding, to let the system autonomously constitutes a unity, distinct from the surroundings (topological closure); (2) ability to completely specify, by molecularly embodied internal rules and operations, the construction and the degradation of all components, without the need of being heterodirected or instructed (organizational closure); (3) ability of exchanging of matter and energy with the surroundings, keeping itself thermodynamically open and continuously functioning out of the equilibrium; (4) possibility to adapt to the external conditions by plastic modification the network of their internal processes while remaining organizationally closed; (5) and possible evolution by the principles of diversification and selection. Such an organization is called *autopoietic* (self-constructing), and it was identified by H. Maturana and F. Varela in the 1970s (Varela et al., 1974). Autopoiesis thus becomes a convenient and elegant theoretical framework to guide the variegate experimental efforts to fabricate synthetic (artificial) cells (SCs/ACs), especially with respect to studies oriented at the origins and emergence of life. Other systemic theories are available, such as the chemoton theory (Ganti, 1975, 2003) and others (Cornish-Bowden and Cárdenas, 2020), but autopoiesis stands out, in our opinion, for its broader and deeper implications.^1^

To date, SC research has definitely put down roots. The number of scholars working in the field is constantly increasing, as well as the number of published articles, often placed in renowned journals. There is an enthusiastic involvement of researchers coming from different backgrounds. New centers, networks, consortia, and initiatives are currently driving the field forward (Schwille et al., 2018; Frischmon et al., 2021; Staufer et al., 2021). Importantly, the construction of *living* SC (considered the “Holy Grail” of the field), the understanding of the non-life to life transition, and the determination of the minimal complexity of living beings, have been flanked by other relevant goals. Pragmatic approaches aiming at the construction of *non-living* SCs are interesting too*,* having advantages such as easier realization and a potentially early use for basic understanding of physiological processed and in biotechnology. Whether or not the goal of SC research refers to living or non-living SCs, such a new “technology” represents a genuine novelty in modern science and constitutes an original and promising platform for investigating theoretical issues as well (Damiano and Stano, 2020; Magarini and Stano, 2021; Stano, 2022).

The construction of different types of cell-like systems with non-trivial complexity is now within the experimental reach. Since several excellent reviews on technical advancements in SCs research have been published recently (Cho and Lu, 2020; Gaut and Adamala, 2021; Ivanov et al., 2021; Lussier et al., 2021), the discussion below will focus, we hope, on less explored subjects, and aims at inspiring future investigation scenarios. Deepening the knowledge and broadening the range of interest on SCs may proceed, according to our viewpoint, according to four tracks: the theoretical, scientific, technological and educational ones.

## The theoretical track: What SCs actually are

Irrespective of the scientific goal behind the construction of SCs (or protocells), the very fact that a cell-like system can be built in controlled laboratory conditions elicits a question about our theoretical/epistemological understanding of what they actually are. Several interesting analyses have discussed the place of SB and SCs in current scientific understanding of life, from historical and epistemological perspectives (Deplazes and Huppenbauer, 2009; Morange, 2009; Moya et al., 2009; Deplazes-Zemp, 2016; Zwart, 2019). The question is not only whether or not SCs can ever be “alive” *hic et nunc*, but whether or not the category of synthetic life is the same of natural life. Moreover, the very rich landscape of approaches, materials, systems that are currently explored makes difficult to define “what SCs are”, and what is the role given to structure, organization, or function in order to evaluate and compare SCs. Attempting to address these and other questions is *per se* a stimulating intellectual journey.

An intriguing interpretation considers SB (and in particular SC research) as the wetware branch of the “*Sciences of the Artificial*” (Cordeschi, 2002; Damiano et al., 2011; Damiano and Stano, 2018). It means that SCs shares with robotics (the hardware branch) and artificial intelligence (AI, the software branch) a common set of scopes, perspectives, and theoretical analyses. These three approaches aim at constructing models that reproduce the biological phenomenology and/or organization, often following the “understanding by building” strategy (Kaneko, 2006). Classical as well as newer concepts related to information and communication theories, computation, self-organization, emergence and complexity can be explored in an extraordinary innovative way by means of SB. These concepts, when properly developed and understood in the SB molecular domain, become new tools for facing long discussed issues like machine/organism dichotomy (Deplazes and Huppenbauer, 2009; Nicholson, 2013) and the related computer/mind one (of course, here we mean minimal organisms with mind-like cognitive features). For example, the functioning of currently studied SCs can be simulated by an algorithm: their behavior is Turing computable. On the other hand, Turing computability of autopoietic (and thus living) systems has been questioned (Letelier et al., 2003; McMullin, 2004). Theoretical investigations related to current and future SCs are quite interesting indeed. If modern SB tools were available to early cyberneticians, the latter would have been certainly interested in them (Wiener et al., 1943; MacKay, 1969).

Let us focus here on the possible contribution of SC research to cognitive sciences, just to make an example. Embodied cognition is one of the three main branches of cognitive sciences, together with classical and connectionistic approaches (Dawson, 2013); it emphasizes the causal perception-action loop that a cognitive agent realizes by interacting with its body (and through its body) in an environment, where it is *situated* (Varela et al., 1992; Shapiro, 2011). This is made possible by sensorimotor capacities embedded in the agent body. As we have recently argued in a dedicated article, SB provides an excellent platform for investigations on “chemical embodied AI” via the development of properly designed wetware models (i.e., SCs) (Damiano and Stano, 2021). For example, in order to model minimal cognition, SCs should cope with environmental perturbation by adaptive mechanisms of self-regulation. It has been proposed to graft *chemical neural networks* in SCs (e.g., based on protein phosphorylation; Gentili and Stano, 2022) that respond to physico-chemical stimuli coming from the surrounding. However, to be adaptive, such networks must be able to self-regulation, and this is not at all trivial to achieve. Nevertheless, the latter seems an easier goal if compared to the rather challenging “whole-SC” autopoiesis (Damiano and Stano, 2018; see also Di Paolo, 2003; Kiverstein et al., 2022).

## The scientific track: Integrate functions to reach higher complexity

This is, perhaps, the most obvious and important direction to look at. Imminent developments in SC research and technology must necessarily face the challenge of constructing systems with higher degree of organization and complexity. In this respect, the *integration* of the several different “modules” available so far in more complex SCs becomes a crucial milestone. A rich and ever increasing repertoire of functional “modules” for SCs operations have been developed in isolated way (e.g., protein synthesis, growth-division, DNA duplication, sending-receiving signals, *etc.*). The integration of these modules can be additive or synergic. For example, constructing SCs made of several “orthogonal” or “insulated” modules would correspond to an additive (linear) increase of complexity, while the combination of interrelated and causally dependent modules would bring about SCs of higher complexity, especially when self-regulatory properties emerge, because the embedded functions are more difficult to disentangle (higher “wholeness”). The first approach leads to an engineered system that can be decomposed into blocks, resembling top-down designed machine mechanisms; the second approach appears more bio-inspired as it points to interwoven processes and organism-like organization.

To face the difficulty of achieving high degrees of integration, an evolutionary approach has been proposed (Abil and Danelon, 2020). Directed evolution strategies should be considered as well (Sakatani et al., 2018; Okauchi and Ichihashi, 2021), especially when connected to adaptive responses. From the *architectural* viewpoint, complexification can be achieved *via* multiple compartmentalization. The latter can be hierarchical, i.e., according to a nested design (Altamura et al., 2021), or referred to 2D or 3D tissue-like systems (Bayley et al., 2019; Dupin et al., 2022): in both cases the behavior of the resulting “whole” will depend on the number, type, and function of constituent compartments.

## The technological track: Looking for practical applications

As mentioned, most of the research on the construction of cell-like systems generally refers to basic scientific questions. However, SC technology is so genuinely innovative that can provide more, and demonstrate its practical utility. A decisive forward leap must come from considering SCs as a biotechnological platform. What are the practical uses of SCs? Who would produce or buy SCs, and why? These questions are often asked when SC research is presented to applied science-oriented audience, and require urgent answers.

The well-established liposome technology for drug delivery and the recent introduction of anti-SARS-CoV-2 vaccines based on RNA-lipid nanoparticles suggest a possible role of SCs as a kind of “smart” drug delivery (or drug producing) agents. The idea of using *ad hoc* designed enzyme-filled particles for enzyme replacement therapy, for instance, is not new at all (Chang, 1972). More recently, LeDuc and collaborators lucidly illustrated a scenario that resonates with SC philosophy (LeDuc et al., 2007). The advancements made on SC communicative properties (Lentini et al., 2017) let us imagine SCs that perceive their environment, and behave in programmable way in biological surroundings (Sato et al., 2022). The pioneer investigations on SCs producing a cancer-killing toxin (Krinsky et al., 2018), or on bacteria-killing SCs that operate upon a bacterial stimulus (Ding et al., 2018) provide a couple of illustrative examples. A realistic discussion about these developments should include however a consideration: the recent trend in SC research focuses on large structures (tens of micrometers), while therapeutic particles planned to be used for systemic administration must be rather small (typically <200 nm). The construction of sophisticated cell-like systems with such small size has been rarely reported (Pereira de Souza et al., 2009; Pols et al., 2019).

Further (and possibly nearer) applications can be devised when SC-like systems are conceived as tools for biotechnological research, exploiting the superior interfacing features between SC and other biological entities, and the possibility of designing SCs with a programmable behavior. For example, SCs could (i) mimic biological cells in viral research; (ii) host membrane sensors and/or reconstituted internal processes which are the target of drug action, to screen drug libraries; (iii) be hybridized with exosomes to complement and/or tailor their properties; (iv) be immobilized in form of biochip in order to respond in complex cell-like manner to several effectors, e.g., to run sophisticated tests; (v) be engineered as virus-like particles for treating cellular cultures or for special transfections; (vi) constitute—together with biological cells—hybrid organoids, or other sort of organized 2D/3D structures, or gel-embedded ensembles.

## The educational track: SCs as a learning topic in “system thinking” programs

Whether or not SCs are designed as primitive cell models, or as non-living biotechnological tools, or as artificial autopoietic systems, it is evident that the very practice of their fabrication must embrace a *systemic* perspective. Systems are those entities or wholes, made of distinct parts, where the relations between the parts count as much as, if not more of, the parts themselves. Static and dynamic orders, patterns, and qualities become central to understanding biological phenomenology and complexity (Capra, 1996). In the case of SCs, the systemic perspective include both structure and organization, in the sense that SC properties, behavior, and features depend on how their components are assembled as a physical unity in space (e.g., due to containment in self-bounding compartments), and on the relations undergoing between the components (e.g., the *in situ* produced *α*-hemolysin chains self-assemble as a heptameric pore on the membrane, allowing small molecules enter or leave the SC lumen (Noireaux and Libchaber, 2004)). A systemic perspective is required for understanding, designing, constructing systems of all types.

While biology students are relatively well acquainted with systemic thinking (e.g., thanks to biochemistry, physiology, and ecology courses), it is not uncommon that students of other disciplines are less familiar with subjects as feedback, homeostasis, autonomy, compartmentation, multiple levels of organization, emergent phenomena, and circular organization^2^. The theory and the practice of SC construction is a convenient and valuable topic for courses on system thinking, as it can provide an opportunity to introduce systemic concepts at any educational level.

Another fecund intersection comes in mind, especially for chemistry students, when we consider the area of *systems chemistry* (Ruiz-Mirazo et al., 2014; Ashkenasy et al., 2017)*.* The focus of systems chemistry goes beyond the mere building of chemical structures, and points to design chemical processes and systems that display features as autocatalysis, self-regulation, reaction-diffusion dynamics and oscillations, out-of-equilibrium dynamics, often exploiting the advantages of micro-compartmentalization. SCs are *de facto* major targets not only for SB, but for systems chemistry too.

## Concluding remarks

This contribution aims at addressing the call made in the Research Topic “Insights in Synthetic Biology 2021*: Novel Developments, Current Challenges, and Future Perspectives*”, that solicited forward-looking contributions describing the future challenges in SB. In particular, the subject of bottom-up SCs has been presented, highlighting its position in SB and its scientific relevance. The four “tracks” described above mirror the interests of the author and do not claim to be exhaustive.

## Footnotes

1 Autopoiesis provides a description of “what life is” entirely in terms of causally concatenated relations of processes and components that (*i*) recursively generate themselves (processes and components) and (*ii*) determine a physical unity, which is physically distinct (and distinguishable) from the environment. Moreover, the autopoietic dynamics self-regulate to compensate the perturbations exerted by the environment, in so far as the induced change can be subordinated to the maintenance of the autopoietic organization (i.e., they are “autonomous”). The allowed perturbations constitute the “cognitive domain” of the unity. Concepts as autonomy, cognition, phenomenology, identity, coherence, structural coupling, plasticity, mind-likeness have roots in the autopoietic dynamics. Autopoiesis contributes to constructivism. Interested readers can further refer to (Varela et al., 1974; Varela, 1979; Maturana and Varela, 1980; Luisi, 2003; Ruiz-Mirazo and Moreno, 2004).

2 By “circular organization” we mean that the system dynamics is constituted by concatenated processes which form, as a whole, a network of causally codependent processes. Such a dynamic organization is constantly regenerated owing to the existence of all processes, as in the case of cellular metabolism.
